# Cancer chemoprevention by oleaster (Elaeagnus angustifoli L.) fruit extract in a model of hepatocellular carcinoma induced by diethylnitrosamine in rats

**DOI:** 10.17179/excli2017-389

**Published:** 2017-07-18

**Authors:** Zahra Amereh, Nasrin Hatami, Farshad H. Shirazi, Saman Gholami, Seyed Hojjat Hosseini, Maryam Noubarani, Mohammad Kamalinejad, Sina Andalib, Fariborz Keyhanfar, Mohammad Reza Eskandari

**Affiliations:** 1 Department of Pharmacology and Toxicology, School of Pharmacy, Zanjan University of Medical Sciences, Zanjan, Iran; 2 Pharmaceutical Sciences Research Center, Shahid Beheshti University of Medical Sciences, Tehran, Iran; 3 Department of Physiology and Pharmacology, School of Medicine, Zanjan University of Medical Sciences, Zanjan, Iran; 4 Department of Pharmacognosy, Faculty of Pharmacy, Shahid Beheshti University of Medical Sciences, Tehran, Iran; 5 Department of Pharmacology, Faculty of Medicine, Iran University of Medical Sciences, Tehran, Iran

**Keywords:** E. angustifolia, cancer prevention, carcinogenesis, liver cancer, Russian olive, wild olive

## Abstract

Hepatocellular carcinoma (HCC) is a frequent and fatal human cancer with poor diagnosis that accounts for over half a million deaths each year worldwide. *Elaeagnus angustifolia *L. known as oleaster has a wide range of pharmacological activities. This study aimed to investigate the chemopreventive effect of aqueous extract of *E. angustifolia *fruit (AEA) against diethylnitrosamine (DEN)-induced HCC in rats. HCC was induced in rats by a single injection of DEN (200 mg/kg) as an initiator. After two weeks, rats were orally administered 2-acetylaminofluorene or 2-AAF (30 mg/kg) as a promoter for two weeks. Oleaster-treated rats were orally pretreated with the increasing doses of AEA two weeks prior to DEN injection that continued until the end of the experiment. In the current study, a significant decrease in serum biomarkers of liver damage and cancer, including alfa-fetoprotein (AFP), gamma glutamyl transpeptidase (GGT), alanine transaminase (ALT), and aspartate transaminase (AST) was observed in AEA-treated rats when compared to HCC rats. Furthermore, the oleaster extract exhibited *in vivo *antioxidant activity by elevating reduced glutathione (GSH) contents as well as preventing lipid peroxidation in the liver tissues of DEN-treated rats. The relative weight of liver, a prognostic marker of HCC, was also reduced in oleaster-treated rats. To conclude, our results clearly demonstrated that oleaster fruit possesses a significant chemopreventive effect against primary liver cancer induced by DEN in rats. It can be suggested that the preventive activity of oleaster against hepatocarcinogenesis may be mediated through the antioxidant, anti-inflammation, and antimutagenic effects of the fruit.

## Introduction

Primary liver cancer or hepatocellular carcinoma (HCC) is the fifth most common malignancy, with 0.25-1 million new cases diagnosed annually in the world (Guo and Wang, 2009[17]; Frau et al., 2013[15]). Currently, there is no proven effective chemotherapy or surgical intervention for HCC and a lot of focus has been put on cancer preventive strategies as an acceptable approach for controlling the HCC incidence. Chemoprevention is one of these strategies, which means the use of natural products, chemicals or their combinations to reduce the risk or delay the onset of cancer (Singh et al., 2014[35]). Chemoprevention interferes with the different stages of carcinogenesis, i.e. initiation, promotion, and progression and decreases the risk of tumor formation. Several strategies have been used for HCC chemoprevention, including immunization against hepatitis B virus (HBV), antiviral therapy in patients with chronic hepatitis B or C virus, prescribing drugs such as statins, and the use of selected dietary agents like coffee, vitamin E and fish oil (Bravi et al., 2009[6]; Chang et al., 2009[7]; Demierre et al., 2005[10]; Lai and Yuen, 2013[21]; Sawada et al., 2012[33]; Singh et al., 2014[35]). In addition, medicinal herbs and phytochemicals play an essential role in HCC chemoprevention and they have become known as a most promising and potentially cost-effective approach to prevent this aggressive cancer (Okano et al., 2011[25]; Stagos et al., 2012[36]).

*Elaeagnus angustifolia *L. (oleaster, Russian olive, wild olive, silver berry) is a medicinal plant from *Elaeagnacea *family. The fruit of *E. angustifolia *has been used in traditional and folklore medicine of different countries for the treatment of various diseases, including rheumatoid arthritis, osteoporosis, asthma, diarrhea, and female aphrodisiac. Oleaster has also been used in Iranian folk medicine as a hepatoprotective and anti-inflammatory agent (Farzaei et al., 2015[13]). Recent findings show that the fruit possesses various pharmacological and therapeutic effects such as antioxidant, anti-inflammation, antitumour, antimutagenic, antibacterial, antifungal, and gastroprotective effects (Amiri Tehranizadeh et al., 2016[3]; Faramarz et al., 2015[12]; Hamidpour et al., 2016[18]; Panahi et al., 2016[28]).

Primary liver cancer is a prototype of inflammation-associated cancer and chronic liver diseases are the most common causes of HCC. Furthermore, free radical formation and oxidative DNA damage have a key role in hepatocellular carcinogenesis (Aravalli et al., 2013[4]). Considering the antioxidant, anti-inflammatory and antimutagenic effects of oleaster; this study was designed to evaluate the chemopreventive effect of aqueous extract of *E. angustifolia *fruit (AEA) against diethylnitrosamine (DEN)-induced hepatocellular carcinoma in rats. DEN is a potent hepatic carcinogen that has extensively been used as an experimental carcinogen in cancer biology research to induce HCC or different types of benign and malignant tumors in rodents (Healy et al., 2015[20]).

## Materials and Methods

### Chemicals

2-Acetylaminofluorene (2-AAF), bovine serum albumin (BSA), thiobarbituric acid, and DEN were purchased from Sigma-Aldrich Co. (Taufkirchen, Germany). All other chemicals were of the highest commercial grade available.

### Preparation of plant extract

*E. angustifolia *fruits were collected from Ardabil Province, Iran. The collected fruits were scientifically approved by the Department of Botany, Shahid Beheshti University of Medical Sciences (SBUMS) and a voucher sample was preserved for reference in SBUMS Herbarium (Voucher number: 1056). Then, the different parts of the fruits, including peel, flesh, and seed were separated, dried, and powdered. After that the extract was prepared by the maceration of the powder (100 g) with 900 mL distilled water for 30 min. Accordingly, the fruit extract was filtered and concentrated to 100 mL, then stored at -20 °C. The moisture levels of the extract were determined by placing 2 g of the final extract in an oven at 60-65 °C for 72 h and were then weighed. Weight loss was used as the moisture indicator. The final extract contained 24 % water, which was dissolved in distilled water at the desired concentrations just before use (Pourahmad et al., 2010[30]).

### Determination of total polyphenol content

Total polyphenol content was determined by spectrophotometry, using gallic acid as the standard based on the Folin-Ciocalteu method as previously described (Gholami et al., 2017[16]).

### Animals

Male Sprague-Dawley rats weighing 180 to 200 g were housed in ventilated plastic cages over PWI 8-16 hardwood bedding. There were 12 air changes per h, 12 h light photoperiods, an environmental temperature of 21-23 °C, and a relative humidity of 50-60 %. The animals were fed a standard normal chow diet and given tap water ad libitum. Principles of laboratory animal care (NIH publication No. 85-23, revised 1985) were followed. All experiments were conducted according to the ethical standards and protocols approved by the Committee of Animal Experimentation of Zanjan University of Medical Sciences, Zanjan, Iran (protocol approval number: A-12-501-24).

### Hepatocarcinogenesis model

The experimental model of HCC was induced in rats by DEN as the initiator and 2-AAF as the promoter of hepatocarcinogenesis according to the protocol described previously with a few modifications (Espandiari et al., 2005[11]). Briefly, rats were fasted for 96 hours and then were refed for 24 hours as a proliferative stimulant. Afterward, rats were injected only a single intraperitoneally (i.p.) dose of DEN (200 mg/kg body weight) for initiating hepatocarcinogenesis. After two weeks, liver cancer development was promoted with a once-daily oral administration of 2-AAF (30 mg/kg body weight) for two weeks. HCC was confirmed by histopathological evaluations and the measurement of liver damage and cancer markers, including alfa-fetoprotein (AFP), gamma glutamyl transpeptidase (GGT), alanine transaminase (ALT), and aspartate transaminase (AST) (Seydi et al., 2016[34]).

### Experimental design 

**Figure 1:**
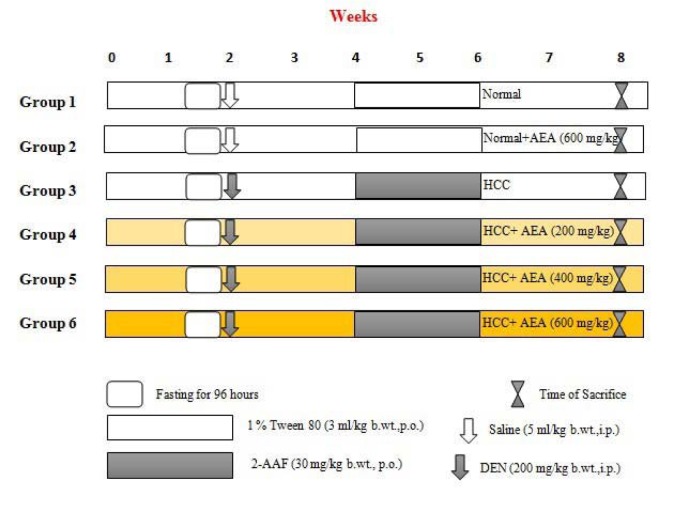
Experimental design. b.wt.: body weight; i.p.: intraperitoneal; p.o.: per os (orally)

As shown in Figure 1(Fig. 1), rats were randomly divided into six different groups of six rats each (n = 6). Group 1 (Control) animals fed with standard diet and served as a normal control, which injected with the single dose of saline. Group 2 were normal animals that treated only with daily oral dose of AEA (600 mg/kg body weight) from the beginning of the experiment. Group 3 (HCC) rats served as untreated HCC animals as described before. Test group animals (Groups 4-6) were pretreated with the increasing doses of AEA (200, 400, and 600 mg/kg body weight, respectively) two weeks prior to DEN injection that continued until the end of the study. The different concentrations of the extract were selected based on previous investigations and were administered orally once daily using an intragastric tube for 8 weeks (Ahmadiani et al., 2000[1]; Wu et al., 2001[40]).

### Sample preparations

On three different time points, animals were anaesthetized by ether inhalation and blood was collected from the orbital sinus and centrifuged at 1500 x g for 20 minutes at 4 °C to obtain serum for biochemical analysis. At the end of the experimental period, body weights of the animals were recorded and rats were sacrificed. Then, the liver tissues were quickly taken, weighed, and frozen for the determination of hepatic oxidative stress markers. Frozen liver samples were homogenized in ice-cold Tris-HCL buffer (150 mM, pH 7.4) (Amin et al., 2011[2]). The relative liver weight was calculated as the percentage ratio of liver weight to the body weight.

### Evaluation of biochemical parameters

Serum AFP is one of the most frequent diagnostic markers for HCC, which was determined by Calbiotech AFP ELISA Kit (Amin et al., 2011[2]). The activities of biochemical markers of liver damage, including GGT, ALT, and AST were determined by commercially available enzyme kits (Pars Azmoon, Tehran, Iran) (Mirmohammadlu et al., 2015[24]). The assays were performed according to the protocols supplied with the kits.

### Determination of protein concentration

The concentration of liver protein was measured by the Coomassie blue protein- binding method using BSA as the standard (Franciscato et al., 2009[14]).

### Determination of hepatic oxidative stress markers

Hepatic GSH contents were estimated in liver homogenate by a spectrophotometric method using dithiobis-2-nitrobenzoic acid (DTNB) as the indicator of GSH and expressed as µg/mg protein. The intensity of the yellow color produced in the samples was recorded at 412 nm with a UV spectrophotometer (Infinite M200, TECAN) (Riener et al., 2002[31]).

The level of malondialdehyde (MDA) as one of the important markers of hepatic lipid peroxidation was estimated in liver homogenate by reading the absorbance of the supernatant layer at 532 nm with an ELISA reader instrument (Infinite M200, TECAN). MDA levels were presented as µg/mg protein (Mihara and Uchiyama, 1978[23]).

### Statistical analysis

The homogeneity of variances was tested using Levene's test. The results were analyzed using one-way analysis of variance (ANOVA) followed by Tukey's HSD as the post hoc test. The data were presented as the mean±SD (n=6). The results with level of significance (*P*<0.05) were regarded as significant.

## Results

### Total phenolic content 

Total phenolic content was found to be 55.12 ± 2.68 mg gallic acid equivalents (GAE) per gram of AEA (mg of GAE/g of plant extract). The given values are mean±SD of three different determinations.

### Effects of AEA on serum biochemical parameters

**Figure 2:**
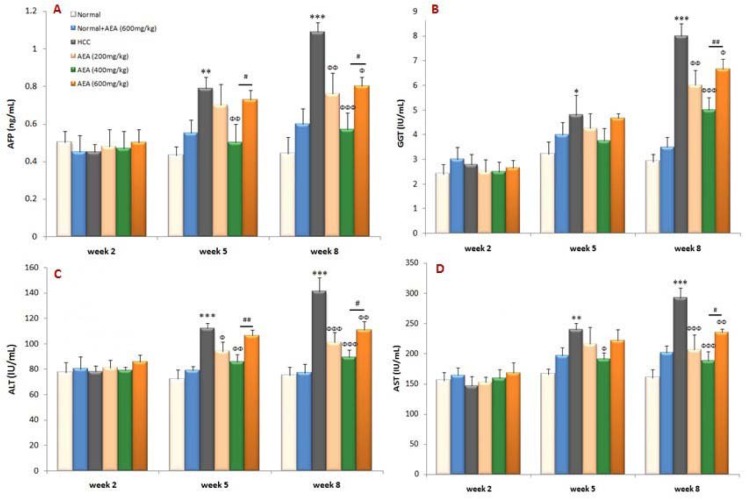
Effects of the oleaster extract (AEA) on serum biochemical parameters: (A) AFP, (B) GGT, (C) ALT, and (D) AST. Values are presented as mean±SD. * *P*<0.05, ** *P*<0.01, *** *P*<0.001 compared to normal group in corresponding time; Ф *P*<0.05, ФФ *P*<0.01, ФФФ *P*<0.001 compared to HCC rats in corresponding time; # *P*<0.05, ## *P*<0.01 compared to the previous concentration

AFP level has been widely used clinically as a tumor marker for HCC that was markedly enhanced in DEN-treated rats compared with normal animals at the end of the experiment (*P*<0.001). As shown in Figure 2(A)(Fig. 2), the elevation in the serum marker of liver cancer was effectively decreased in AEA-treated rats. In the present study, the activities of GGT, ALT, and AST were also significantly increased in HCC rats as compared to normal rats. As can be seen in Figure 2(B-D)(Fig. 2), the oral pretreatment of different concentrations of AEA to HCC rats caused a significant reduction in the serum markers of liver injury. Interestingly, AEA at the concentration of 400 mg/kg body weight daily acted more powerful than the other concentrations in attenuating the markers of liver damage and cancer (Figure 2(Fig. 2)). It can be also seen that the oral administration of AEA (600 mg/kg body weight/day) to normal rats (Group 2) did not change the biochemical markers (Figure 2(Fig. 2)).

### Effects of AEA on the hepatic markers of oxidative stress

**Figure 3:**
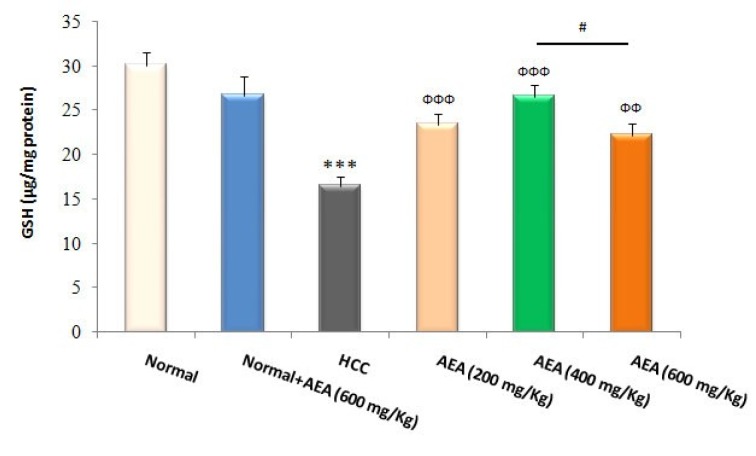
Effects of the oleaster extract (AEA) on hepatic GSH contents. Values are presented as mean±SD. *** *P*<0.001 compared to normal group; ФФ *P*<0.01, ФФФ *P*<0.001 compared to HCC rats; # *P*<0.05 compared to the previous concentration

**Figure 4:**
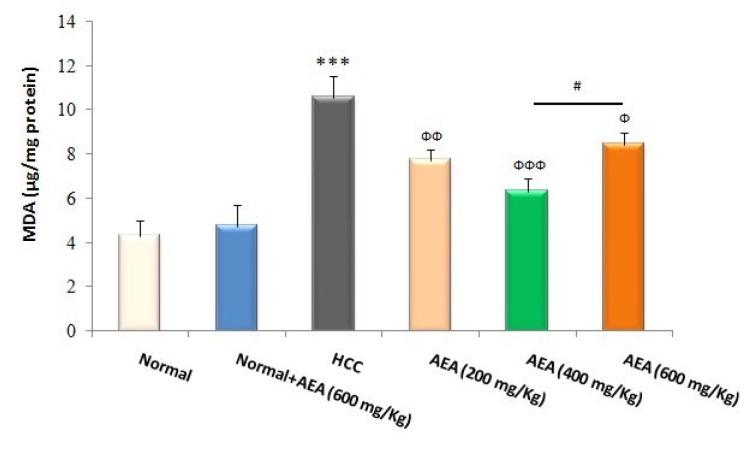
Effects of the oleaster extract (AEA) on liver lipid peroxidation. MDA formation as the marker of lipid peroxidation was expressed as µg/mg protein. Values are presented as mean±SD. *** *P*<0.001 compared to normal group; Ф *P*<0.05, ФФ *P*<0.01, ФФФ *P*<0.001 compared to HCC rats; # *P*<0.05 compared to the previous concentration

GSH and its related antioxidant defense system are required to neutralize free radicals such as electrophilic carcinogens and to counteract oxidative stress. As presented in Figure 3(Fig. 3), liver GSH levels were reduced in HCC rats compared to normal animals (*P*<0.001). Moreover, MDA levels as a reliable marker of lipid peroxidation were increased in the livers of DEN-treated rats compared with those of normal rats (*P*<0.001) (Figure 4(Fig. 4)). In the current study, the oral administration of the oleaster extract to HCC rats caused a rise in GSH levels as well as a fall in lipid peroxidation (Figures 3(Fig. 3), 4(Fig. 4)). Again, the concentration of 400 mg/kg body weight/day was more effective than the other concentrations in decreasing the hepatic oxidative stress markers (Figures 3(Fig. 3), 4(Fig. 4)). It was also shown that the treatment of normal rats (Group 2) with AEA (600 mg/ kg body weight daily) did not alter the oxidative stress markers (Figures 3(Fig. 3), 4(Fig. 4)).

### Effects of AEA on relative liver weight

**Figure 5:**
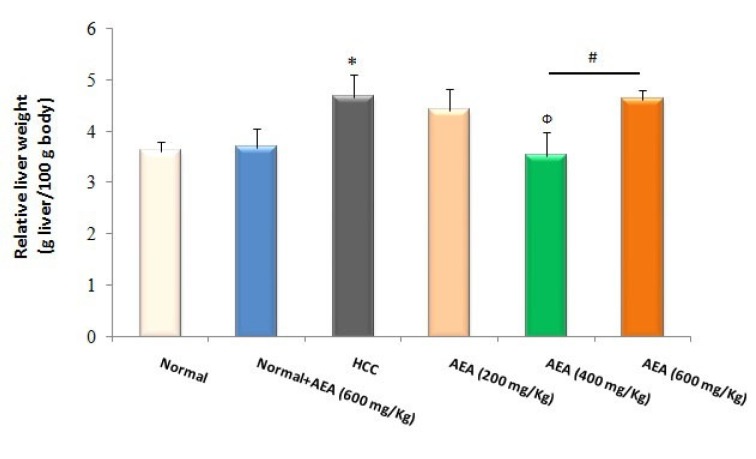
Effects of the oleaster extract (AEA) on relative liver weight. Values are presented as mean±SD. * *P*<0.05 compared to normal group; Ф *P*<0.05 compared to HCC rats; # *P*<0.05 compared to the previous concentration

As indicated in Figure 5(Fig. 5), the relative liver weight of HCC rats was increased compared with normal animals at the end of the study (*P*<0.05). In addition, the pretreatment of AEA (400 mg/kg body weight daily) to HCC rats caused a significant decrease in relative liver weight (*P*<0.05). Again, the oral administration of AEA (600 mg/kg body weight/ day) to normal rats (Group 2) did not affect the relative liver weight (Figure 5(Fig. 5)).

## Discussion

This is the first report showing chemo-preventive effect of oleaster fruit in DEN-induced hepatocellular carcinoma in rats. In this study, the pretreatment of oleaster to HCC rats caused a remarkable reduction in the serum markers of liver damage and cancer. Serum AFP level is the golden standard among diagnostic markers for HCC (Chou et al., 2017[8]; Park et al., 2017[29]).

AFP is a glycoprotein that is synthesized during early foetal life and after birth its serum concentration falls quickly. It has been suggested that AFP acts as a transport molecule for many ligands such as heavy metals, bilirubin, and many xenobiotics. Furthermore, AFP has a role in the regulation of cell proliferation and an immunosuppressive activity (Terentiev and Moldogazieva, 2006[37]). Elevated AFP serum levels are only seen in certain tumors (e.g. HCC), non-tumoral conditions (e.g. cirrhosis), and maternal serum during pregnancy. In the current investigation, the elevated level of AFP was markedly decreased by the oleaster extract.

GGT, ALT, and AST are also the most widely used HCC tumor markers. GGT is a cell surface enzyme that involves in glutathione metabolism. Its main function is to sustain cysteine levels in the body to conserve intracellular homeostasis of oxidative stress. GGT expression in tumor cells provides a selective advantage to the cells during tumor promotion and enables them to preserve elevated levels of intracellular GSH. Therefore, it has been suggested that GGT is an independent prognostic indicator in patients with HCC (Sadeeshkumar et al., 2016[32]; Wu et al., 2016[39]). Data in our study indicated that the oral administration of oleaster to DEN- treated rats reduced GGT, ALT, and AST activities.

Glutathione and glutathione-related enzymes play a pivotal role in the metabolism and detoxification of free radicals and carcinogenic compounds. They are also involved in the regulation of carcinogenic mechanisms and cancer cell death (Ortega et al., 2011[27]). It has been reported that GSH levels and the activities of GSH-dependent enzymes in the liver tissues of HCC patients were decreased due to the impairment of antioxidant defense system (Czeczot et al., 2006[9], Lee et al., 2007[22]). Our results clearly demonstrated that the oleaster extract counteracted DEN-induced oxidative stress and restored GSH levels in the liver of oleaster-treated rats.

Lipid peroxidation, a reliable marker of oxidative stress, is a well-defined mechanism of cellular damage in different pathological conditions. MDA is the end product of lipid peroxidation, which is implicated in the promotion and progression stages of carcinogenesis (Onlom et al., 2017[26]). As presented in Figure 4(Fig. 4), hepatic MDA levels were excellently reduced by the oleaster fruit extract. Finally, our results showed that the oleaster extract prevented the increase in relative liver weight as a prognostic marker of HCC.

The fruit of oleaster has high nutritional values and contains essential amino acids, carbohydrates, minerals, and vitamins A, C, E, and K (Farzaei et al., 2015[13]). In addition, a wide variety of bioactive compounds have been identified in *E. angustifolia *fruit involving in its broad spectrum of pharmacological effects. Oleaster fruit contains different kinds of phenolic acids and flavonoids such as 4- hydroxybenzoic acid, 4-hydroxycinnamic acid, benzoic acid, caffeic acid, and isorhamnetin (Farzaei et al., 2015[13]). Dietary phenolics or polyphenols are secondary metabolites of plants found in different kinds of fruits and vegetables. It has been demonstrated that the different classes of polyphenols such as flavonoids and phenolic acids have numerous benefits for human health and a protective role against various diseases (Tsao, 2010[38]). In recent years, there has been a considerable interest in their potential therapeutic use as chemopreventive agents. Several *in vitro *and *in vivo *studies have shown that plant polyphenols possess protective effects against the development and growth of liver cancer suggesting that these natural compounds are promising candidate agents for HCC chemoprevention. They reverse, suppress or prevent the different steps of hepatocarcinogenesis through various mechanisms (Stagos et al., 2012[36]). In the present study, the extract was prepared from whole fruit and more than 5 % of the total weight related to polyphenols. Therefore, it can be concluded that the chemopreventive activity of oleaster may be, at least partly, mediated through the anti-carcinogenic properties of polyphenolic compounds.

It is strongly believed that inflammation- mediated processes and oxidative stress contribute to the development and progression of hepatocarcinogenesis through different mechanisms (Bishayee et al., 2010[5]; He and Lin, 2017[19]). Also, it has been reported that oleaster fruit possesses potent antioxidant and anti-inflammatory effects as well as antimutagenic and antitumour activities (Amiri Tehranizadeh et al., 2016[3]; Faramarz et al., 2015[12]; Hamidpour et al., 2016[18]). Therefore, it can be suggested that these biochemical properties are responsible, at least partly, for the chemoprevention of the fruit. On the other hand, further studies to find out mechanisms underlying the effect would be complementary to this study. The results of this study demonstrate that bioactive constituents from oleaster would be valuable for their use in the chemoprevention of liver cancer. However, we believe that further studies are required to elucidate what active compounds are responsible for the chemopreventive effect of this valuable fruit and to detect synergy among the different components of oleaster.

In conclusion, our results showed that oleaster has a chemopreventive effect against DEN-induced hepatocarcinogenesis in rats and this valuable fruit can be proposed as a promising candidate for HCC chemoprevention.

## Conflict of interest

The authors do not declare any conflict of interest.

## Acknowledgement

This study was funded by a Research Grant from Zanjan University of Medical Sciences, Deputy of Research (Grant No: A-12-501-24).
